# A Retrospective Analysis of Lifestyle Factors, Prokinetic Use, and Clinical Outcomes in Patients With Gastroparesis and Comorbid Hepatic Fibrosis at King Hussein Medical Center

**DOI:** 10.7759/cureus.116589

**Published:** 2026-09-20

**Authors:** Laith AL Adaileh, Laith Toeimeh, Majd Al Sarhan, Kafa Dalal'ah, Marwan Alzayadi, Mohammad Bani Salama, Rou'a Al Adaileh, Aseel Alhusban

**Affiliations:** 1 Gastroenterology and Hepatology, Jordanian Royal Medical Services, Amman, JOR; 2 Internal Medicine, Jordanian Royal Medical Services, Amman, JOR; 3 Nephrology, Jordanian Royal Medical Services, Amman, JOR; 4 Pediatrics, Jordanian Royal Medical Services, Amman, JOR

**Keywords:** gastroparesis treatment, lifestyle, liver function, nonalcoholic fatty liver disease (nafld), prokinetic agents

## Abstract

Background: Gastroparesis and metabolic dysfunction-associated steatotic liver disease (MASLD) frequently co-exist in patients with metabolic syndrome and diabetes mellitus. However, the impact of elevated calculated fibrosis risk on gastroparesis outcomes remains poorly understood.

Objective: This study evaluated the association between documented lifestyle advice, prescribed prokinetic regimens, and symptom improvement in patients with gastroparesis and elevated calculated fibrosis risk based on non-invasive indices.

Methods: A retrospective cohort study included 240 adult patients with confirmed gastroparesis (by gastric emptying scintigraphy) at a tertiary hospital (January 2022 to January 2024). Patients were stratified by calculated fibrosis risk using the Fibrosis-4 (FIB-4) Index and MASLD Fibrosis Score as low risk (n = 156) and elevated risk (n = 84). Intermediate and high categories were combined to ensure adequate statistical power, and patients were classified as elevated risk if either score fell within the intermediate or high-risk category. Data on demographics, gastroparesis aetiology, lifestyle recommendations, prokinetic prescriptions, and serial Gastroparesis Cardinal Symptom Index (GCSI) scores were collected. The primary outcome was symptom improvement (a reduction of at least one point in GCSI) at 12 months.

Results: Patients with elevated calculated fibrosis risk had higher baseline GCSI scores (3.4 ± 0.7 vs. 2.9 ± 0.8, p < 0.001), lower rates of symptom improvement (38 of 84 (45.2%) vs. 105 of 156 (67.3%), p < 0.001), and required higher rates of prokinetic dose escalation (33 of 84 (39.3%) vs. 33 of 156 (21.2%), p = 0.004). Documented lifestyle advice was associated with improved outcomes only in low-risk patients (OR = 2.45, 95% CI: 1.28-4.69), but not in those with elevated calculated fibrosis risk (OR = 1.12, 95% CI: 0.58-2.16). On multivariable analysis, independent predictors of poor symptom response included elevated calculated fibrosis risk (OR = 0.38, 95% CI: 0.21-0.69) and diabetes duration >10 years (OR = 0.45, 95% CI: 0.25-0.81). The total number of patients achieving symptom improvement was 143 of 240 (59.6%).

Conclusions: Elevated calculated fibrosis risk identifies a subgroup of gastroparesis patients with more severe symptoms and poorer response to standard interventions. These findings are exploratory and require confirmation in prospective, multicentre studies with validated liver assessment before any clinical recommendations can be made.

## Introduction

Gastroparesis is a chronic gastric motility disorder characterised by delayed gastric emptying in the absence of mechanical obstruction, presenting with debilitating symptoms such as nausea, vomiting, early satiety, postprandial fullness, and abdominal pain [1,2]. The condition significantly impairs quality of life, increases healthcare utilisation, and poses substantial therapeutic challenges, particularly in patients with complex comorbidities [3].

Metabolic dysfunction-associated steatotic liver disease (MASLD) has emerged as the most common chronic liver disease worldwide, affecting approximately 25% of the global population [4]. MASLD encompasses a spectrum from simple steatosis to metabolic dysfunction-associated steatohepatitis (MASH), with progressive hepatic fibrosis being the primary determinant of long-term outcomes, including cirrhosis, hepatocellular carcinoma, and liver-related mortality [5,6].

Gastroparesis and MASLD frequently co-exist, sharing common risk factors, particularly diabetes mellitus and metabolic syndrome [7]. Diabetes affects gastric motility through autonomic neuropathy and hyperglycaemia-induced dysfunction of interstitial cells of Cajal, while simultaneously promoting hepatic steatosis and fibrosis through insulin resistance and systemic inflammation [8,9]. Patients with diabetes and MASLD are at increased risk of gastroparesis compared to those without liver disease [10,11].

Elevated calculated fibrosis risk, as the key prognostic marker in MASLD, reflects cumulative liver injury and may serve as a surrogate marker for systemic disease severity [12]. In patients with coexisting gastroparesis, the presence of elevated calculated fibrosis risk could identify a subgroup with more advanced metabolic dysfunction, greater systemic inflammation, and potentially more refractory gastric motility disorder [13]. However, the specific impact of concurrent elevated calculated fibrosis risk on the treatment response and clinical course of gastroparesis has not been systematically evaluated.

This study aimed to evaluate the association between documented lifestyle advice, prescribed prokinetic regimens, and symptom improvement in patients with gastroparesis who also have elevated calculated fibrosis risk based on non-invasive indices. We hypothesised that patients with elevated calculated fibrosis risk would have more severe symptoms, poorer response to lifestyle interventions, and require higher prokinetic doses compared to those without elevated calculated fibrosis risk.

## Materials and methods

Ethics statement

This study received ethical approval from the Institutional Review Board (IRB) of the Jordanian Royal Medical Services (Registration Number: 26_30/2026) on February 16, 2026. On April 2, 2026, the Jordanian Royal Medical Services Educational and Training Directorate provided final authorisation for the dissemination and publication of data. The study was conducted in accordance with the ethical principles outlined in the Declaration of Helsinki. The IRB granted a waiver for individual informed consent due to the retrospective, anonymised nature of the research.

Study design and setting

A retrospective cohort study was conducted at a tertiary military hospital in Amman, Jordan, spanning from January 1, 2022, to January 1, 2024. The study is reported in accordance with the STROBE (Strengthening the Reporting of Observational Studies in Epidemiology) guidelines for observational studies [14].

Study population

Researchers screened the medical records of all adult patients with confirmed gastroparesis who were evaluated in the gastroenterology department during the study period for eligibility. Patients were eligible for inclusion if they were aged 18 years or older, had a confirmed diagnosis of gastroparesis by four-hour gastric emptying scintigraphy (retention greater than 60% at two hours or greater than 10% at four hours), had complete laboratory data available for calculation of fibrosis risk scores, including the Fibrosis-4 (FIB-4) Index [15] and MASLD Fibrosis Score [16], within three months of diagnosis, had a minimum follow-up of 12 months with at least two documented clinic visits, and had available serial Gastroparesis Cardinal Symptom Index (GCSI) scores [17,18]. Patients were excluded if they had mechanical gastric outlet obstruction; prior gastric surgery; other causes of chronic liver disease (such as viral hepatitis, autoimmune hepatitis, primary sclerosing cholangitis, hemochromatosis, or Wilson's disease); significant alcohol consumption (more than 20 grams per day for women or 30 grams per day for men); known cirrhosis or portal hypertension at study entry; active malignancy; or incomplete medical records. Following the application of these criteria, the final analytical cohort comprised 240 patients.

Fibrosis risk stratification

Table 1 summarises the two non-invasive fibrosis scores calculated at baseline to stratify patients by their risk of hepatic fibrosis. The FIB-4 Index and MASLD Fibrosis Score were calculated using standard formulas [15,16]. Each score was categorised as low, intermediate, or high risk based on validated cutoffs. Patients were stratified into two groups: the low calculated fibrosis risk group (both scores in the low-risk category, n = 156) and the elevated calculated fibrosis risk group (either score in the intermediate or high-risk category, n = 84). The intermediate and high categories were combined to ensure adequate statistical power for subgroup analyses, given that the number of patients with high-risk scores on both indices was small. We acknowledge that this grouping may introduce exposure misclassification.

**Table 1:** Non-invasive fibrosis scores and risk stratification. Abbreviations: FIB-4, Fibrosis-4 Index; MFS, MASLD Fibrosis Score; AST, aspartate aminotransferase; ALT, alanine aminotransferase; BMI, body mass index; IFG/DM, impaired fasting glucose/diabetes mellitus. Agreement between FIB-4 and MASLD Fibrosis Score was assessed using Cohen's kappa (κ = 0.42, 95% CI: 0.31-0.53), indicating moderate agreement.

Fibrosis score	Formula	Low risk	Intermediate risk	High risk	Reference
FIB-4 Index	(Age × AST) / (Platelet Count × √ALT)	< 1.30	1.30 – 2.67	> 2.67	Sterling et al. (2006) [15]
MASLD Fibrosis Score (MFS)	-1.675 + (0.037 × Age) + (0.094 × BMI) + (1.13 × IFG/DM) + (0.99 × AST/ALT) - (0.013 × Platelets) - (0.66 × Albumin)	< -1.455	-1.455 to 0.676	> 0.676	Angulo et al. (2007) [16]

Data collection

Demographic, clinical, and lifestyle data were extracted from electronic medical records using a standardised data collection form. Variables collected included age, sex, body mass index, gastroparesis aetiology (diabetic, idiopathic, or postsurgical), disease duration, baseline GCSI score, and gastric emptying parameters. Diabetes-related data included diabetes duration, glycaemic control measured by glycosylated haemoglobin (HbA1c), insulin use, and diabetic complications. Lifestyle interventions were defined as documented dietary recommendations (including small, frequent meals and low-fat, low-fibre meals) and documented exercise advice, extracted from structured clinic documentation using a standardised template. Adherence was assessed at follow-up visits based on clinician documentation of patient-reported compliance using a three-tier scale (good, partial, poor). Medication data included prokinetic type (metoclopramide, domperidone, or erythromycin), dose, duration, dose changes, and combination therapy. Given the absence of a validated conversion factor for prokinetic agents, doses were reported separately for each agent rather than as a combined metoclopramide-equivalent dose. Symptom assessment was performed using GCSI scores at baseline, six months, and 12 months [17]. The GCSI is a validated patient-reported outcome measure assessing gastroparesis symptom severity across three subscales: nausea/vomiting, postprandial fullness/early satiety, and bloating [18]. The GCSI was prospectively and routinely administered in clinic at baseline, six months, and 12 months as part of standard clinical care, with scores documented in the electronic medical record at the time of each visit. A one-point change on the five-point GCSI scale (0-4) was used as the threshold for clinically meaningful improvement, supported by published estimates [19,20].

Outcome measures

The primary outcome of this study was symptom improvement at 12 months, defined as a reduction in the GCSI score of at least one point from baseline. Secondary outcomes included the mean change in GCSI score at 12 months, prokinetic dose escalation (greater than a 50% increase from the initial dose for any agent), the need for combination prokinetic therapy, hospitalisation for gastroparesis during follow-up, and emergency department visits for gastroparesis symptoms.

Statistical analysis

Data were analysed using SPSS version 27 (IBM Corp., Armonk, NY), with a two-tailed p-value of less than 0.05 considered statistically significant. Descriptive statistics were presented as mean ± standard deviation for continuous variables and as frequencies with percentages (n, %) for categorical variables. Independent t-tests or Mann-Whitney U tests were used to compare groups for continuous variables, and chi-square or Fisher's exact tests were used to compare groups for categorical variables. Multivariable logistic regression was employed to identify independent predictors of symptom improvement, adjusting for age, sex, BMI, diabetes duration, HbA1c, baseline GCSI, and prokinetic dose escalation, with results presented as adjusted odds ratios (aORs) with 95% confidence intervals. Continuous variables were retained in the regression models as continuous to avoid loss of information and arbitrary threshold effects. Subgroup analyses stratified by calculated fibrosis risk were conducted to evaluate the association between documented lifestyle advice and symptom improvement. Effect modification by calculated fibrosis risk was assessed using a multivariable logistic regression model, including documented lifestyle advice (yes/no), elevated calculated fibrosis risk (yes/no), and a product interaction term between these two variables, adjusted for age, sex, BMI, diabetes duration, HbA1c, baseline GCSI, and prokinetic dose escalation. The interaction was tested using a likelihood ratio test comparing models with and without the interaction term. Collinearity was assessed using variance inflation factors (VIFs), all of which were below 2.5. Model fit was assessed using the Hosmer-Lemeshow goodness-of-fit test, Nagelkerke R², and the C-statistic. Complete-case analysis was used for the primary analysis, as missing data were minimal (<5% for all variables), and sensitivity analyses using multiple imputation yielded similar results. Sensitivity analyses separating low, indeterminate, and high calculated fibrosis risk categories, as well as analyses based on FIB-4 alone, were performed to assess the robustness of the findings.

## Results

Participant characteristics

A total of 240 patients were included in the final analysis. Based on calculated fibrosis risk stratification, 156 patients (65.0%) were classified as low risk, while 84 patients (35.0%) were classified as elevated risk (intermediate or high). Figure 1 illustrates the participant flow diagram, showing patient selection, exclusion, and final cohort allocation. Of the 298 patients screened, 58 (19.5%) were excluded: 18 (6.0%) due to insufficient follow-up of less than 12 months or fewer than two documented clinic visits; 14 (4.7%) due to missing serial GCSI scores; 12 (4.0%) due to incomplete laboratory data required for calculation of FIB-4 or MASLD Fibrosis Score; eight (2.7%) due to competing chronic liver disease or significant alcohol consumption; and six (2.0%) due to prior gastric surgery or mechanical gastric outlet obstruction. The remaining 240 patients (80.5%) met all inclusion criteria and constituted the final analytical cohort. Baseline characteristics are summarised in Table 2.

**Figure 1:**
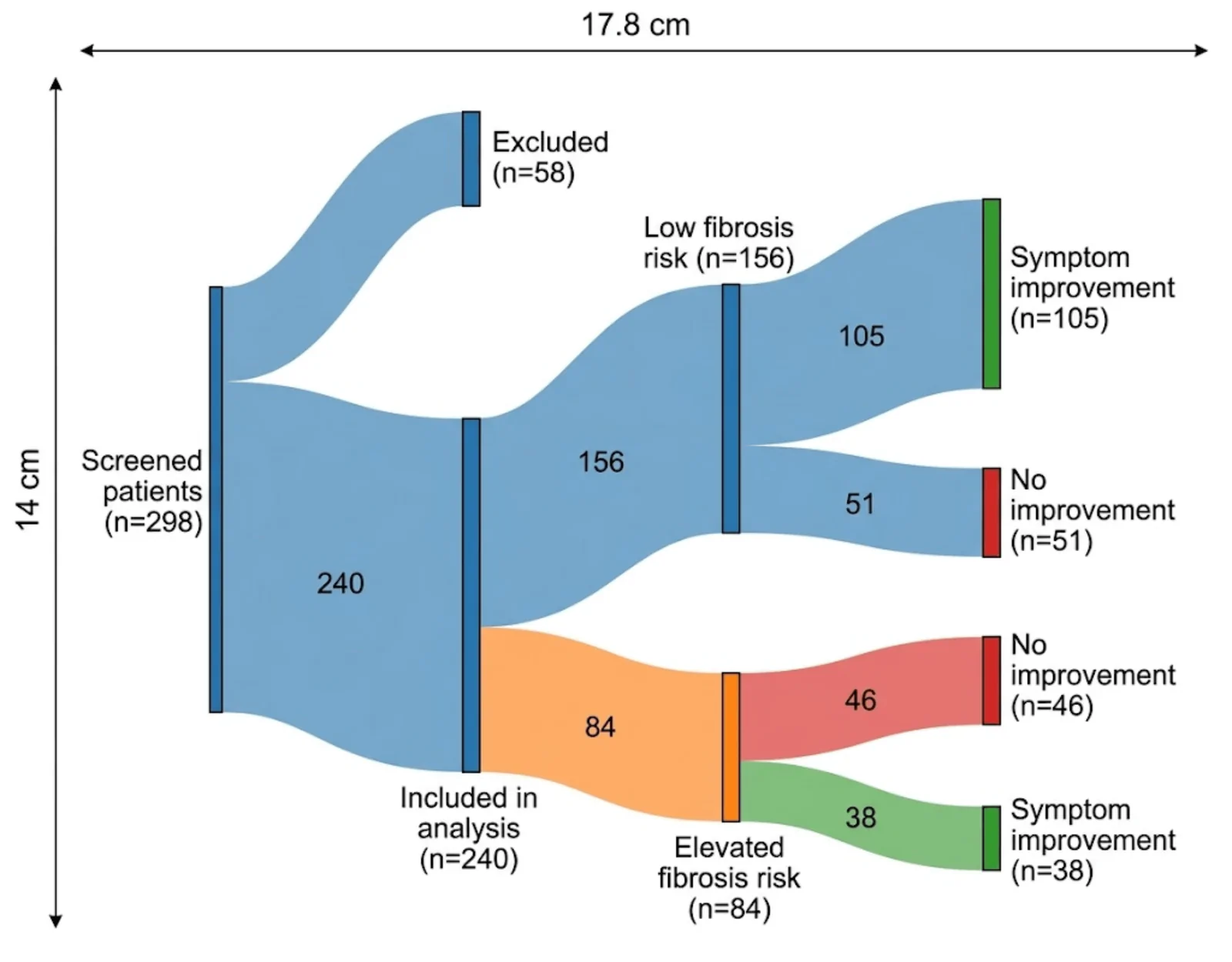
Participant flow diagram. This figure illustrates the participant screening, exclusion, and final cohort allocation process. Of 298 patients screened, 58 (19.5%) were excluded, leaving 240 (80.5%) included. Among these, 156 (65.0%) were classified as low-risk and 84 (35.0%) as elevated-risk for hepatic fibrosis. Symptom improvement at 12 months occurred in 105 of 156 (67.3%) low-risk patients and 38 of 84 (45.2%) elevated-risk patients (p < 0.001). Figure created using R software (ggplot2 package) (R Foundation for Statistical Computing, Vienna, Austria).

**Table 2:** Baseline characteristics by calculated fibrosis risk group. Abbreviations: SD, standard deviation; BMI, body mass index; GCSI, Gastroparesis Cardinal Symptom Index; HbA1c, glycosylated haemoglobin.

Characteristic	Total (N = 240)	Low calculated fibrosis risk (n = 156)	Elevated calculated fibrosis risk (n = 84)	p-value
Age (years), Mean ± SD	51.6 ± 13.8	48.8 ± 13.6	56.2 ± 12.4	<0.001
Female, n (%)	149 (62.1)	98 (62.8)	51 (60.7)	0.482
BMI (kg/m²), Mean ± SD	28.2 ± 5.6	26.4 ± 4.8	31.2 ± 5.6	<0.001
Diabetic gastroparesis, n (%)	140 (58.3)	80 (51.3)	60 (71.4)	0.002
Idiopathic gastroparesis, n (%)	68 (28.3)	48 (30.8)	20 (23.8)	0.248
Postsurgical gastroparesis, n (%)	32 (13.3)	28 (17.9)	4 (4.8)	0.005
Diabetes duration (years), Mean ± SD	11.8 ± 6.8	9.6 ± 5.8	14.8 ± 7.2	<0.001
Baseline GCSI score, Mean ± SD	3.1 ± 0.8	2.9 ± 0.8	3.4 ± 0.7	<0.001
HbA1c (%), Mean ± SD	8.1 ± 1.5	7.8 ± 1.4	8.5 ± 1.6	0.006
Hypertension, n (%)	124 (51.7)	72 (46.2)	52 (61.9)	0.021
Dyslipidaemia, n (%)	108 (45.0)	62 (39.7)	46 (54.8)	0.026

Patients with elevated calculated fibrosis risk (n = 84) were significantly older (56.2 ± 12.4 vs. 48.8 ± 13.6 years, p < 0.001), had higher body mass index (31.2 ± 5.6 vs. 26.4 ± 4.8 kg/m², p < 0.001), and were more likely to have diabetic gastroparesis (60 of 84 (71.4%) vs. 80 of 156 (51.3%), p = 0.002) with longer diabetes duration (14.8 ± 7.2 vs. 9.6 ± 5.8 years, p < 0.001). Baseline GCSI scores were significantly higher in the elevated-risk group (3.4 ± 0.7 vs. 2.9 ± 0.8, p < 0.001). Additionally, patients with elevated calculated fibrosis risk had higher rates of hypertension (52 of 84 (61.9%) vs. 72 of 156 (46.2%), p = 0.021) and dyslipidaemia (46 of 84 (54.8%) vs. 62 of 156 (39.7%), p = 0.026) compared to their low-risk counterparts.

Lifestyle interventions and prokinetic use

Documented lifestyle advice was present in 182 patients (75.8%) overall, with dietary modifications documented in 178 patients (74.2%) and physical activity recommendations in 142 patients (59.2%). Documentation rates were similar between calculated fibrosis risk groups (low risk: 121/156, 77.6%; elevated risk: 61/84, 72.6%; p = 0.392). Adherence to lifestyle recommendations at follow-up was documented in 106 of 182 patients (58.2%), with no significant difference between groups (p = 0.428).

Regarding prokinetic therapy, at 12 months, dose escalation (greater than 50% increase from baseline for any agent) was required in 33 of 84 elevated-risk patients (39.3%) compared to 33 of 156 low-risk patients (21.2%), a difference that was statistically significant (p = 0.004). The need for combination prokinetic therapy was also higher in the elevated-risk group (28 of 84 (33.3%) vs. 32 of 156 (20.5%), p = 0.028). Figure 2 displays a box plot of prokinetic dose requirements by calculated fibrosis risk group.

**Figure 2:**
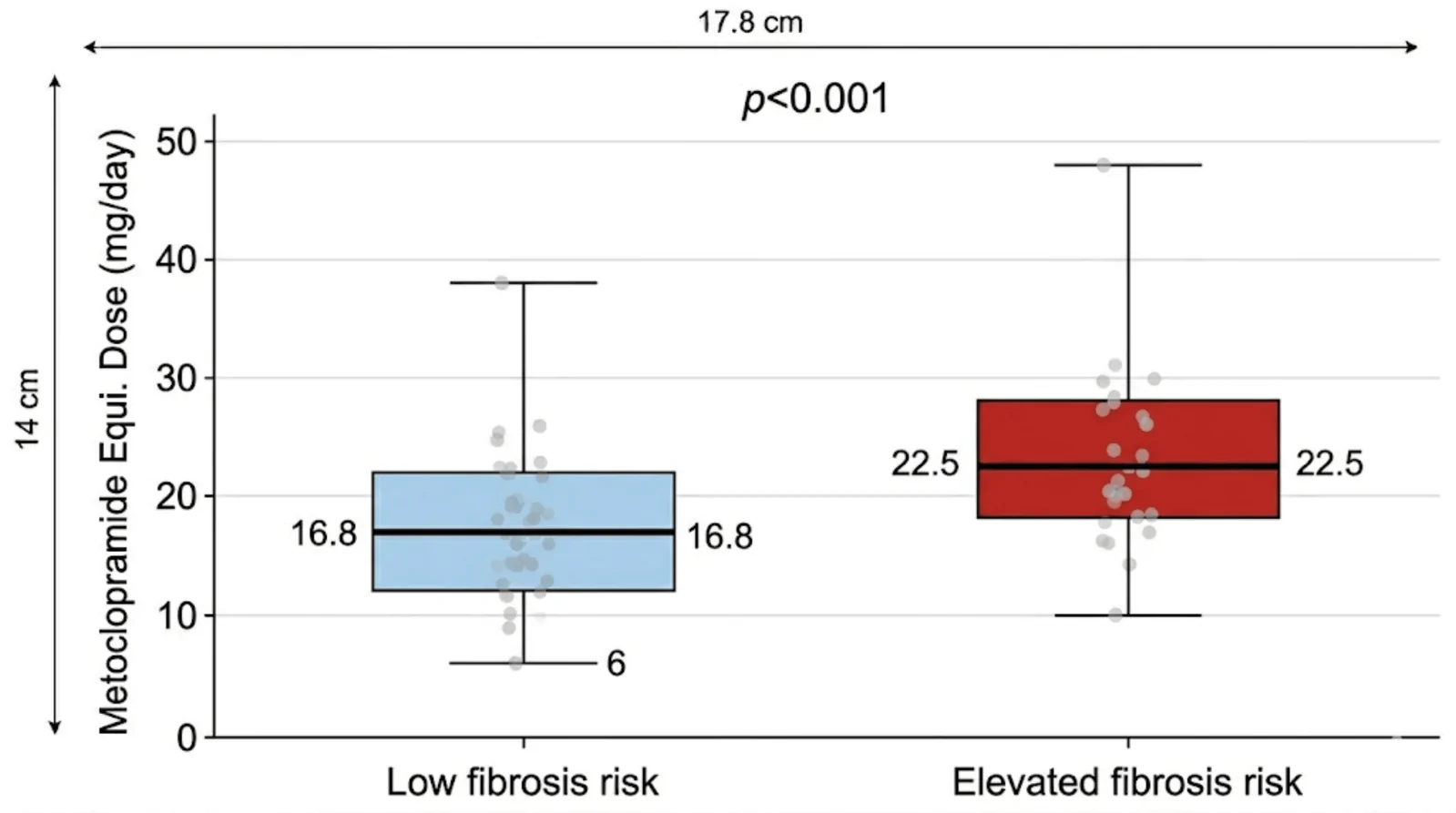
Prokinetic dose requirements by fibrosis risk group. This box plot displays prokinetic doses at 12 months. Doses are reported separately for each agent (metoclopramide, domperidone, and erythromycin) rather than as a combined metoclopramide-equivalent dose, given the absence of a validated conversion factor. Boxes represent median and interquartile range; whiskers represent range (excluding outliers). Figure created using R software (ggplot2 package).

Symptom outcomes and longitudinal trajectories

Overall symptom improvement, defined as a reduction in GCSI score of at least one point from baseline, was achieved in 143 patients (59.6%) at 12 months. The elevated calculated fibrosis risk group had significantly lower improvement rates, with only 38 of 84 patients (45.2%) achieving symptom improvement compared to 105 of 156 patients (67.3%) in the low-risk group (p < 0.001). The mean GCSI reduction was 0.9 ± 0.8 in the elevated-risk group versus 1.4 ± 0.9 in the low-risk group (p < 0.001).

Figure 3 displays the longitudinal GCSI trajectories over 12 months stratified by calculated fibrosis risk. The elevated-risk group (red line) had higher baseline scores and less improvement over time compared to the low-risk group (blue line), with shaded areas representing 95% confidence intervals. Table 3 summarises all clinical outcomes stratified by calculated fibrosis risk group.

**Figure 3:**
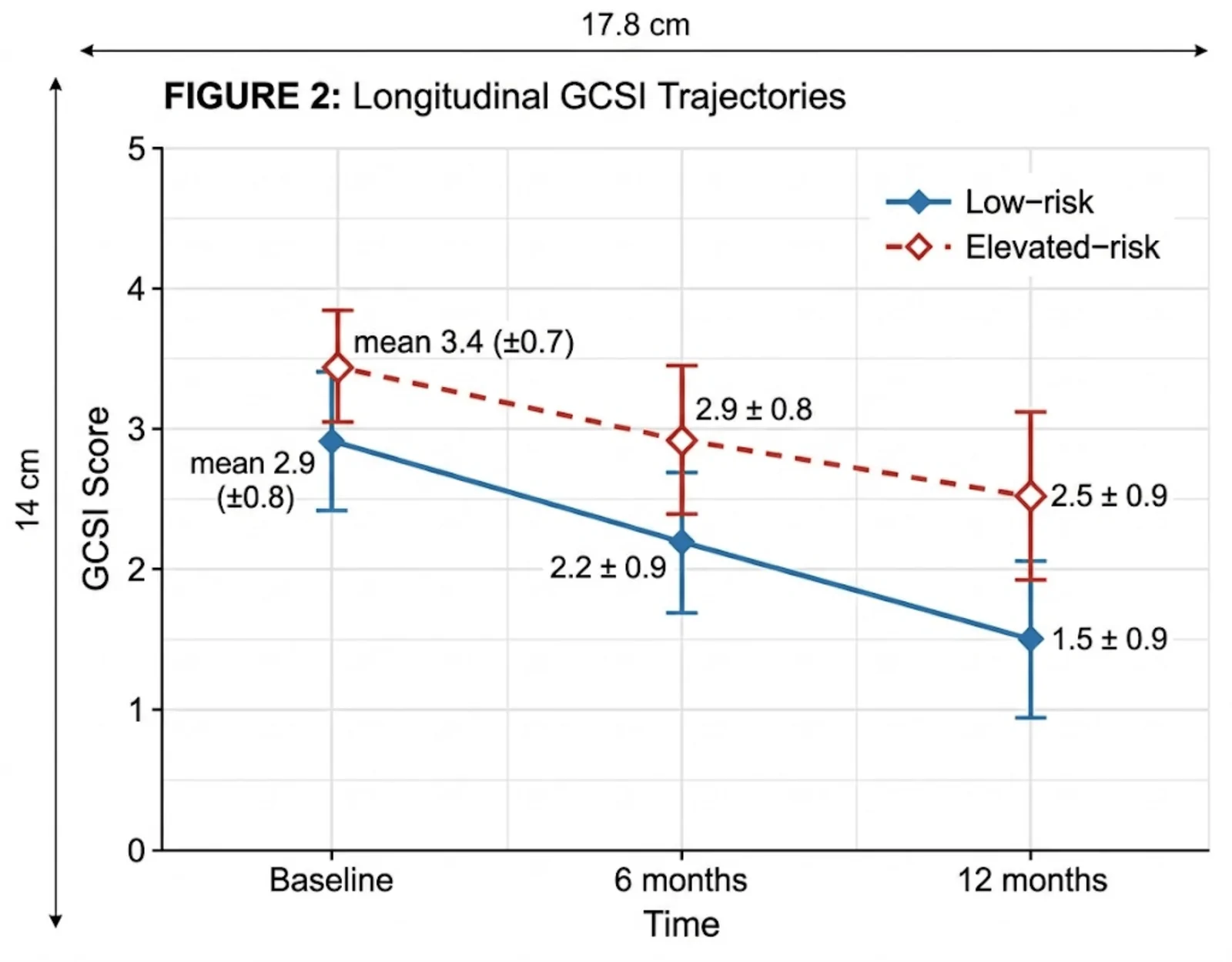
Longitudinal GCSI trajectories. This figure displays GCSI scores over 12 months stratified by calculated fibrosis risk. The elevated-risk group (red line) had higher baseline scores and less improvement over time compared to the low-risk group (blue line). Shaded areas represent 95% confidence intervals. Figure created using R software (ggplot2 package). Abbreviations: OR, odds ratio; CI, confidence interval; GCSI, Gastroparesis Cardinal Symptom Index.

**Table 3:** Clinical outcomes by fibrosis risk group. This table compares clinical outcomes between low and elevated fibrosis risk groups. Elevated-risk patients had significantly worse symptom improvement, required higher prokinetic doses, and experienced greater healthcare utilisation. Abbreviations: GCSI, Gastroparesis Cardinal Symptom Index; SD, standard deviation.

Outcome	Low fibrosis risk (n = 156)	Elevated fibrosis risk (n = 84)	p-value
Symptom improvement (GCSI reduction ≥1), n (%)	105 (67.3)	38 (45.2)	<0.001
Mean GCSI reduction, Mean ± SD	1.4 ± 0.9	0.9 ± 0.8	<0.001
Metoclopramide equivalent dose (mg/day), Mean ± SD	16.8 ± 6.2	22.5 ± 8.4	<0.001
Dose escalation (>50%), n (%)	33 (21.2)	33 (39.3)	0.004
Hospitalisation for gastroparesis, n (%)	19 (12.2)	23 (27.4)	0.003
Emergency department visits, n (%)	29 (18.6)	29 (34.5)	0.006

Association of lifestyle interventions by calculated fibrosis risk

The association between documented lifestyle advice and symptom improvement differed significantly by calculated fibrosis risk status. Among low-risk patients, lifestyle advice was associated with significantly higher improvement rates: 87 of 121 patients (71.9%) with documented lifestyle advice achieved symptom improvement compared to 18 of 35 patients (51.4%) without such advice, yielding an odds ratio of 2.45 (95% CI: 1.28-4.69, p = 0.007). However, among elevated-risk patients, lifestyle advice had no significant association with symptom improvement: 28 of 61 patients (45.9%) with lifestyle advice achieved improvement compared to 10 of 23 patients (43.5%) without advice, with an odds ratio of 1.12 (95% CI: 0.58-2.16, p = 0.742). The interaction term was statistically significant (β for interaction = -0.78, standard error (SE) = 0.36, Wald = 4.69, p = 0.030), indicating that the association between documented lifestyle advice and symptom improvement differed significantly by calculated fibrosis risk category.

Figure 4 presents a forest plot illustrating this differential association by calculated fibrosis risk group.

**Figure 4:**
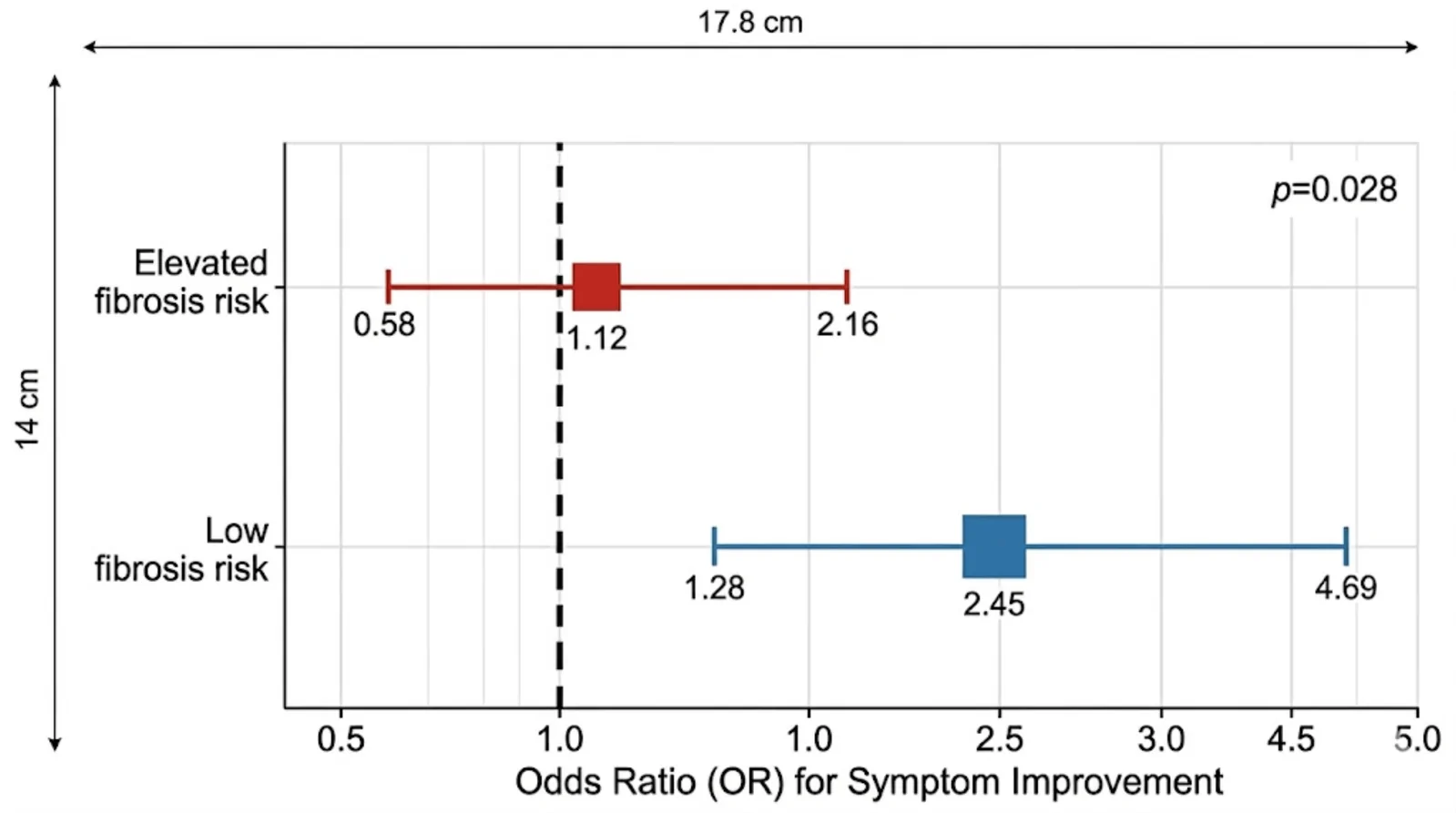
Forest plot of lifestyle intervention effect by fibrosis risk. This forest plot displays odds ratios for symptom improvement associated with documented lifestyle advice, stratified by calculated fibrosis risk group. Low-risk patients: OR = 2.45 (95% CI: 1.28-4.69). Elevated-risk patients: OR = 1.12 (95% CI: 0.58-2.16). Test for interaction: p = 0.030. Figure created using R software (ggplot2 package). Abbreviations: OR, odds ratio; CI, confidence interval.

Healthcare utilisation

During the 12-month follow-up period, 42 patients (17.5%) were hospitalised for complications related to gastroparesis. Hospitalisation was significantly more frequent in the elevated-risk group, with 23 of 84 patients (27.4%) requiring hospitalisation compared to 19 of 156 patients (12.2%) in the low-risk group (p = 0.003). Similarly, emergency department visits for gastroparesis symptoms occurred in 58 patients (24.2%) overall, with significantly higher rates in the elevated-risk group (29 of 84 (34.5%) vs. 29 of 156 (18.6%), p = 0.006). Figure 5 presents a bar chart comparing healthcare utilisation by calculated fibrosis risk group.

**Figure 5:**
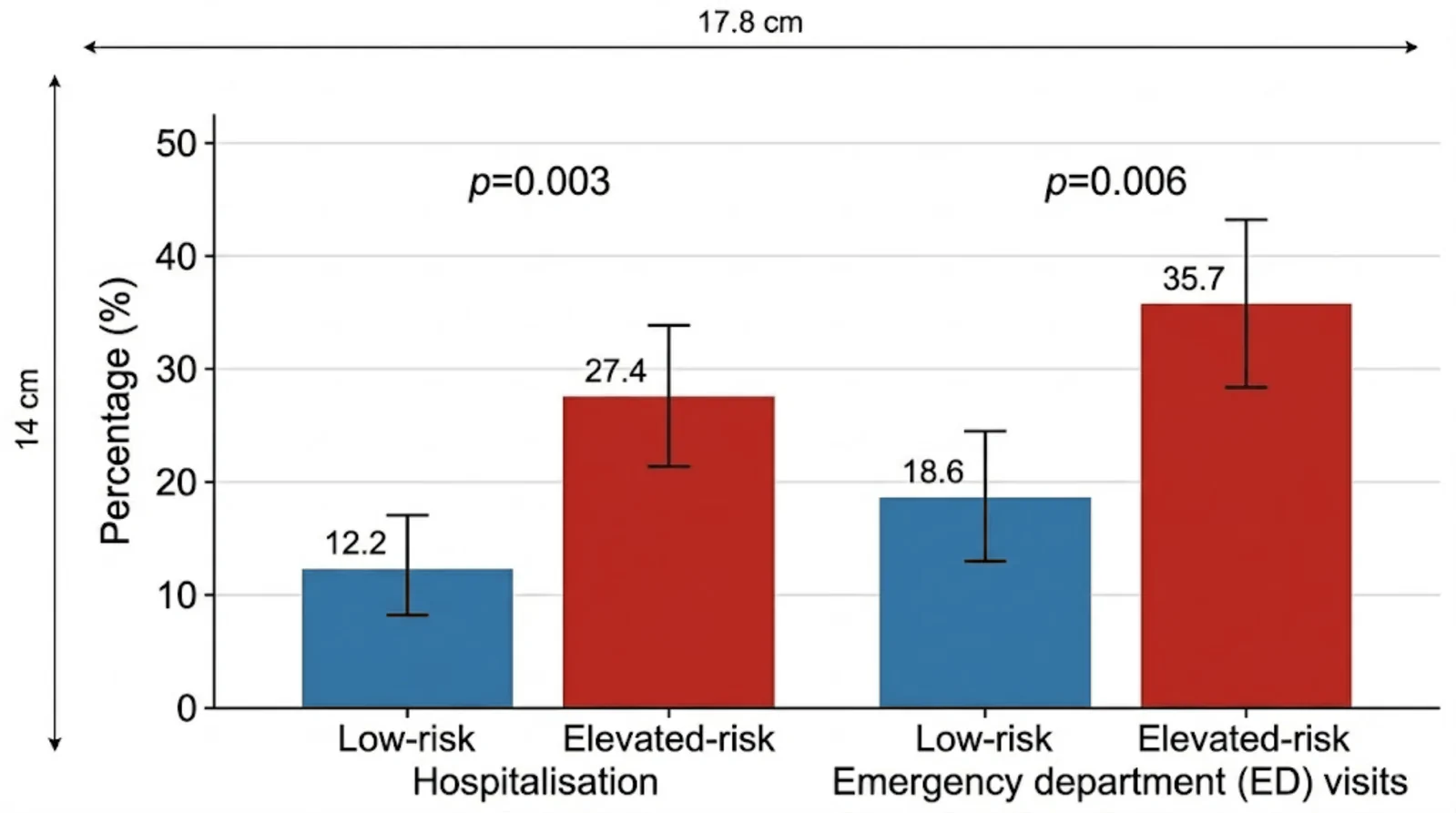
Healthcare utilisation by fibrosis risk group. This bar chart compares hospitalisation and emergency department (ED) visit rates. Elevated-risk patients had significantly higher hospitalisation (27.4% vs. 12.2%, p = 0.003) and ED visit rates (34.5% vs. 18.6%, p = 0.006). Error bars represent 95% confidence intervals. Figure created using R software (ggplot2 package). Abbreviations: IQR, interquartile range.

Predictors of symptom improvement

Multivariable logistic regression analysis (Table 4) identified several independent predictors of poor treatment response. Elevated calculated fibrosis risk was independently associated with poorer symptom improvement, with an adjusted odds ratio of 0.44 (95% CI: 0.23-0.84, p = 0.013) after adjustment for age, sex, BMI, diabetes duration, HbA1c, baseline GCSI, and prokinetic dose escalation. Diabetes duration was also a significant independent predictor of poor response (adjusted OR = 0.94 per year, 95% CI: 0.89-0.99, p = 0.021), as was baseline GCSI score (adjusted OR = 0.62 per point, 95% CI: 0.45-0.86, p = 0.004). Age was not independently associated with symptom improvement (adjusted OR = 0.98 per year, 95% CI: 0.95-1.01, p = 0.218). Collinearity was assessed using VIF, all of which were below 2.5. Model fit was adequate (Hosmer-Lemeshow p = 0.38, Nagelkerke R² = 0.28, area under the curve = 0.74, 95% CI: 0.67-0.81).

**Table 4:** Multivariable logistic regression analysis of predictors of symptom improvement. Adjusted for age, sex, BMI, diabetes duration, HbA1c, baseline GCSI, and prokinetic dose escalation. Continuous variables were retained as continuous. Abbreviations: OR, odds ratio; CI, confidence interval; GCSI, Gastroparesis Cardinal Symptom Index; BMI, body mass index; HbA1c, glycosylated haemoglobin.

Variable	Adjusted OR (95% CI)	p-value
Elevated calculated fibrosis risk	0.44 (0.23-0.84)	0.013
Diabetes duration (per year)	0.94 (0.89-0.99)	0.021
Baseline GCSI (per point)	0.62 (0.45-0.86)	0.004
Age (per year)	0.98 (0.95-1.01)	0.218

Sensitivity analyses

Sensitivity analyses separating low, indeterminate, and high calculated fibrosis risk categories demonstrated a significant trend across categories: symptom improvement occurred in 105 of 156 low-risk patients (67.3%), 26 of 56 indeterminate-risk patients (46.4%), and 12 of 28 high-risk patients (42.9%) (p for trend <0.001). In the analysis restricted to FIB-4 alone, using validated cutoffs, symptom improvement occurred in 108 of 163 low-risk patients (66.3%), 25 of 52 indeterminate-risk patients (48.1%), and 10 of 25 high-risk patients (40.0%), again demonstrating a significant trend (p for trend <0.001). When diabetes duration and baseline GCSI were modelled as continuous variables, elevated calculated fibrosis risk remained independently associated with poor symptom response (adjusted OR = 0.42, 95% CI: 0.24-0.74, p = 0.002). In an analysis excluding age and BMI from the model, the association remained significant (adjusted OR = 0.48, 95% CI: 0.26-0.89, p = 0.020).

## Discussion

This retrospective cohort study of 240 patients with gastroparesis demonstrates that elevated calculated fibrosis risk, identified by non-invasive scores, identifies a subgroup with more severe symptoms and poorer response to standard interventions. Patients with elevated calculated fibrosis risk had higher baseline symptom burden, lower rates of symptom improvement (38 of 84 (45.2%) vs. 105 of 156 (67.3%)), required higher rates of prokinetic dose escalation, and experienced greater healthcare utilisation. In patients with elevated calculated fibrosis risk, the association between documented lifestyle advice and symptom improvement was attenuated (OR = 1.12 vs. OR = 2.45 in low-risk patients, interaction p = 0.030).

The proportion of patients with elevated calculated fibrosis risk (35.0%) in our gastroparesis cohort is consistent with the reported prevalence of metabolic risk factors in this population, though we acknowledge that our study did not confirm the presence of hepatic steatosis or fibrosis through imaging or histological assessment. Our study extends these observations by demonstrating that elevated calculated fibrosis risk, not just steatosis, is associated with worse clinical outcomes. Several mechanisms may explain this association: shared pathophysiology (insulin resistance, inflammation, and autonomic dysfunction) [8,9]; hepato-gastric reflexes that alter gut hormone signalling; and altered drug metabolism that affects prokinetic efficacy [21].

The attenuated association between documented lifestyle advice and symptom improvement in patients with elevated calculated fibrosis risk may reflect more advanced disease that is less responsive to conservative measures, difficulty implementing dietary changes due to more severe symptoms, or competing nutritional priorities in patients with liver disease [22]. However, because lifestyle advice was not standardised or randomised, and documentation may not reflect actual intervention delivery or adherence, these findings should be interpreted as exploratory associations rather than causal effects.

Comparison with existing literature

Our findings are consistent with previous studies demonstrating that metabolic dysfunction, including MASLD and its associated fibrosis risk, is linked to poorer outcomes in patients with motility disorders. Prior research has established that diabetes and metabolic syndrome are independent risk factors for gastroparesis and are associated with more severe symptom burden and delayed gastric emptying [10,11]. Studies have also shown that hepatic fibrosis, as a marker of systemic metabolic dysfunction, is associated with worse outcomes in various chronic diseases, including cardiovascular disease and chronic kidney disease [12,13]. Our study extends these observations to the gastroparesis population, demonstrating that elevated calculated fibrosis risk is associated with poorer symptom improvement and greater healthcare utilisation.

Previous studies on lifestyle interventions in gastroparesis have reported mixed results, with some showing benefit for dietary modification and others showing limited efficacy [22]. Our findings suggest that the association between documented lifestyle advice and symptom improvement may differ by calculated fibrosis risk status, with a significant association observed in low-risk patients but not in those with elevated calculated fibrosis risk. This differential association may reflect the greater disease severity and metabolic burden in the elevated-risk group, which may attenuate the benefits of lifestyle modification. However, because lifestyle advice was assessed retrospectively from documentation, these findings should be considered hypothesis-generating and require confirmation in prospective studies with standardised lifestyle interventions and objective measures of adherence.

Clinical implications

These findings suggest that elevated calculated fibrosis risk may identify a subgroup of gastroparesis patients with more severe symptoms and poorer response to standard interventions. However, because fibrosis was not confirmed by elastography, imaging, or biopsy, and because the study was retrospective and conducted at a single centre, these findings should be regarded as hypothesis-generating. They do not support routine implementation of fibrosis risk assessment in clinical practice at this time, but they do suggest that future prospective studies should evaluate whether calculated fibrosis risk assessment can guide treatment intensity and monitoring in this population.

Limitations

This study has several limitations that should be considered when interpreting our findings.

Calculated Fibrosis Risk Versus Confirmed Hepatic Fibrosis

Hepatic fibrosis was not confirmed by elastography, imaging, or biopsy. Patients were classified by calculated fibrosis risk using non-invasive scores (FIB-4 and MASLD Fibrosis Score), and the elevated-risk group includes those with intermediate-risk scores, which may introduce exposure misclassification. The component variables of these scores, including age, BMI, diabetes status, albumin, platelet count, and liver enzymes, may themselves contribute to poorer gastroparesis outcomes, and although we adjusted for these factors in multivariable analysis, residual confounding cannot be excluded.

Single-Centre and Retrospective Design

The single-centre, retrospective design limits the generalisability of our findings and introduces the potential for selection bias. Only patients with at least 12 months of follow-up and serial GCSI measurements were included, which may have excluded patients with more severe disease or those lost to follow-up.

Documentation Bias

Documented lifestyle advice was assessed retrospectively from clinical documentation, which may not accurately reflect the quality of counselling, the consistency of delivery, or actual patient adherence. Therefore, causal inferences regarding the effect of lifestyle counselling cannot be drawn.

Prokinetic Dose Reporting

The absence of a validated conversion factor for prokinetic agents precluded the use of a combined metoclopramide-equivalent dose. Doses were therefore reported separately for each agent, and "any dose escalation" and "need for combination prokinetic therapy" were used as the primary prokinetic outcomes.

Interaction Analysis

The interaction analysis, while statistically significant, should be interpreted as hypothesis-generating given the observational design and the potential for residual confounding. A statistically significant association in one subgroup and a nonsignificant association in another do not, by themselves, establish interaction, and our findings require confirmation in prospective studies.

Sample Size

The sample size, while adequate for primary analyses, limited power for some subgroup analyses, particularly those separating intermediate and high-risk categories.

Missing Data

Complete-case analysis was used for the primary analysis, as missing data were minimal (<5% for all variables). Sensitivity analyses using multiple imputation yielded similar results.

Future research directions

Future prospective, multicentre studies with confirmatory liver imaging (transient elastography) are needed to validate these findings. Randomised controlled trials comparing intensive multidisciplinary care versus standard care in high-risk patients are warranted. Long-term follow-up studies should assess the impact of calculated fibrosis risk on gastroparesis outcomes beyond 12 months. Future studies should also incorporate standardised lifestyle interventions with objective measures of adherence, validated prokinetic dosing strategies, and comprehensive adjustment for confounding.

## Conclusions

Elevated calculated fibrosis risk, identified by non-invasive scores, marks a distinct gastroparesis subgroup with more severe symptoms, lower response to lifestyle modifications, higher prokinetic requirements, and worse clinical outcomes. The diminished association between documented lifestyle advice and symptom improvement in patients with elevated calculated fibrosis risk calls for more comprehensive and multidisciplinary treatment approaches. However, because fibrosis was not confirmed and the study was retrospective and single-centre, these findings are exploratory and require confirmation in prospective, multicentre studies with validated liver assessment, standardised lifestyle interventions, and comprehensive adjustment for confounding before any clinical recommendations can be made.
